# Landscapes of recovery from psychosis: Co-creating real-world solutions in Urban settings

**DOI:** 10.1192/j.eurpsy.2025.303

**Published:** 2025-08-26

**Authors:** L. A. A. Empson, O. Söderström, M. Winz, J. Favrod, P. Golay, A. Ruggeri, P. Conus

**Affiliations:** 1 Service of General Psychiatry, Department of Psychiatry, CHUV, Treatment and early Intervention in Psychosis Program (TIPP); 2 Faculty of Biology and Medicine, University of Lausanne, Lausanne; 3 Geography Institute, Neuchâtel University, Neuchâtel; 4 SO-PSY, Goumoens-la-Ville; 5 School of Nursing Sciences, La Source University of Applied Sciences and Arts, Lausanne, Switzerland

## Abstract

**Introduction:**

Recovery-oriented care requires a paradigm shift from a vulnerability to a protection model. However, protective factors and resources to recovery in urban milieus remain poorly understood. Whether material, emotional or social, the identification of those resources calls for user-led initiatives and a more situated understanding of environments, where those recovery trajectories occur.

**Objectives:**

The Lausanne-based Urban Remediation program is a multi-stakeholder (service users, psychiatrists, geographers, community actors and public authorities) initiative, which aims to identify key elements of an ‘urban recovery milieu for psychosis’ and create such a milieu in the city of Lausanne (Switzerland). This talk describes the participatory methods used to create a strategy to foster recovery from psychosis in cities to better inform city’s mental health plan and policies.

**Methods:**

We implement a living lab approach aimed at real-world experimentation in four phases: (i) *exploration*, (ii) *co*-*creation*, (iii) *experimentation*, and (iv) *implementation.* During phase one, we’ve used participatory mapping, go-along interviews and photovoice for an *in-situ* engagement with 10 young patients to ensure a systematic understanding of obstacles and resources for recovery. For phase 2, qualitative analysis and collective workshops with the various stakeholders were used to co-elaborate relevant urban interventions and identify partners for further implementation.

**Results:**

Introducing a Living Lab methodology to experiment the recovery-oriented strategy in a limited area in a real-world setting provides us with solutions, which can be further scaled up to inform the creation of a more inclusive city. Lessons learnt with early psychosis patients can benefit to the community as a whole, as high sensitivity of psychotic patients can teach us a great deal both regarding urban stressors and resources common to the general population.

**Conclusions:**

Using real-world methodologies in cities allows to mobilize actors and resources beyond individual resilience to support recovery trajectories. Consistent transdisciplinary efforts are needed to involve all stakeholders (urban planners, mental health plan developers and society at large) for effective user-based changes and implementation of sustainable solutions.

**Disclosure of Interest:**

None Declared

